# Aqua­(1,4,7,10-tetra­aza­cyclo­dodeca­ne)zinc(II) bis­(perchlorate)

**DOI:** 10.1107/S2414314621003977

**Published:** 2021-04-20

**Authors:** Yoshimi Ichimaru, Koichi Kato, Hiromasa Kurosaki, Haruto Fujioka, Misa Sakai, Yoshihiro Yamaguchi, Jin Wanchun, Kirara Sugiura, Masanori Imai, Tohru Koike

**Affiliations:** a College of Pharmacy, Kinjo Gakuin University, 2-1723 Omori, Moriyamaku, Nagoya, Aichi, 4638521, Japan; b Laboratory of Organic Medicinal Chemistry, Faculty of Pharmacy & Pharmaceutical Sciences, Fukuyama University, Fukuyama 729-0292, Japan; c Environmental Safety Center, Kumamoto University, 39-1 Kurokami 2-Chome, Chuo-ku, Kumamoto, 8608555, Japan; d Department of Functional Molecular Science, Institute of Biochemical & Health Sciences, Hiroshima University, Hiroshima 734-8553, Japan; Katholieke Universiteit Leuven, Belgium

**Keywords:** crystal structure, zinc(II) complex, cyclen

## Abstract

The cationic Zn^II^ part of aqua­(1,4,7,10-tetra­aza­cyclo­dodeca­ne)zinc(II) bis­(perchlorate), [Zn(C_8_H_20_N_4_)(H_2_O)](ClO_4_)_2_, exhibits a slightly distorted square-pyramidal coordination environment with a water mol­ecule in the apical position. In the crystal, the macrocyclic ring alternates between two conformations with equal occupancies. Two of the three perchlorate anions are situated about a twofold rotation axis, and one of them shows disorder of the O atoms with occupancies of 0.62 (7) and 0.38 (7). In the crystal, the complexes are connected by inter­molecular hydrogen bonding *via* the perchlorate anions.

## Abstract 2

The cationic Zn^II^ part of aqua­(1,4,7,10-tetra­aza­cyclo­dodeca­ne)zinc(II) diperchlorate, [Zn(C_8_H_20_N_4_)H_2_O](ClO_4_)_2_, exhibits a slightly distorted square-pyramidal coordination environment with a water mol­ecule in the apical position.

## Graphical abstract

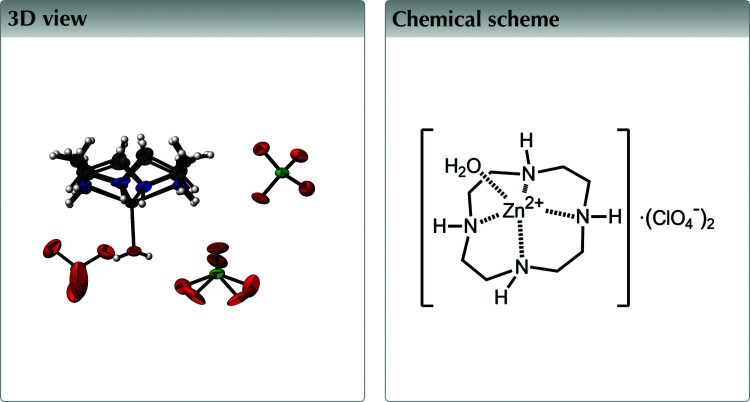

## Structure description

**Figure 1:**
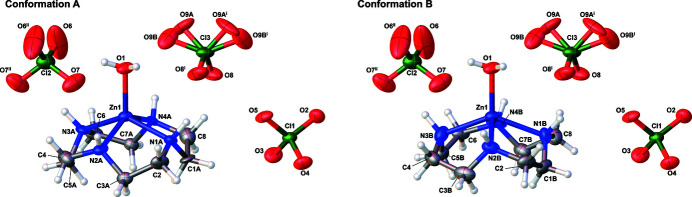
The structures of the molecular entities within the title complex showing 50% displacement ellipsoids. [Symmetry codes: (i) −*x* + 1, *y*, −*z* + 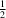 ; (ii) −*x*, *y*, −*z* + 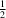 ].

The title complex, [Zn(C_8_H_20_N_4_)H_2_O](ClO_4_)_2_, comprises a cationic Zn^II^ complex and three perchlorate anions, two of which are located about a twofold rotation axis with one of them disordered [occupancy ratio for the corresponding O atoms is 0.62 (7):0.38 (7)]. The macrocyclic ring is disordered, and two alternate conformations of each N–C–C–N bridge can be observed (conformation *A* and *B*) (Fig. 1 ▸), in which four carbon atoms (C2, C4, C6, and C8) are shared. The central Zn^II^ cation is ligated by four N atoms of 1,4,7,10-tetra­aza­cyclo­dodecane (cyclen) in the basal plane, with a Zn^II^-bound H_2_O mol­ecule occupying the apical position. Addison *et al.* (1984 ▸) proposed the geometry index [τ = (*β* − *α*)/60°] to determine if the five-coordinate atom has a square-pyramidal or trigonal–pyramidal coordination environment. The bond angles *β* and *α* are the largest and second-largest in the coordination sphere, respectively; an ideal square pyramid and an ideal trigonal bipyramid have τ = 0 and 1, respectively. In conformation *A*, the N—Zn^II^—N bond angles *α* and *β* are 138.2 (3)° and 138.7 (3)°, respectively; the corresponding bond angles in conformation *B* are 137.4 (4)° and138.7(4)°. The *τ* values are 0.008 and 0.022 for conformations *A* and *B*, respectively. Therefore, the coordination geometry around the central Zn^II^ cation can be described as slightly distorted square-pyramidal. The occupancies for the non-hydrogen atoms of cyclen except for the four carbon atoms (C2, C4, C6, and C8) were set to 0.50. Atom Zn1 is 0.755 (5) and 0.763 (3) Å above the basal plane formed by four N atoms in conformations *A* and *B*, respectively. The Zn1—O1 bond length [1.9721 (4) Å] is within the typical range [1.94–2.03 Å] for similar five-coordinated Zn complexes (Bazzicalupi *et al.*, 1995 ▸; Chen *et al.*, 1994 ▸; Kato & Ito, 1985 ▸; Koike *et al.*, 1994 ▸; Murthy & Karlin, 1993 ▸; Schrodt *et al.*; 1997 ▸). In addition, the mean Zn1—N bond length (2.13 Å) in the title complex is similar to that in the crystal structure of [Zn(cyclen)EtOH](ClO_4_)_2_ (Schrodt *et al.*, 1997 ▸).

**Figure 2:**
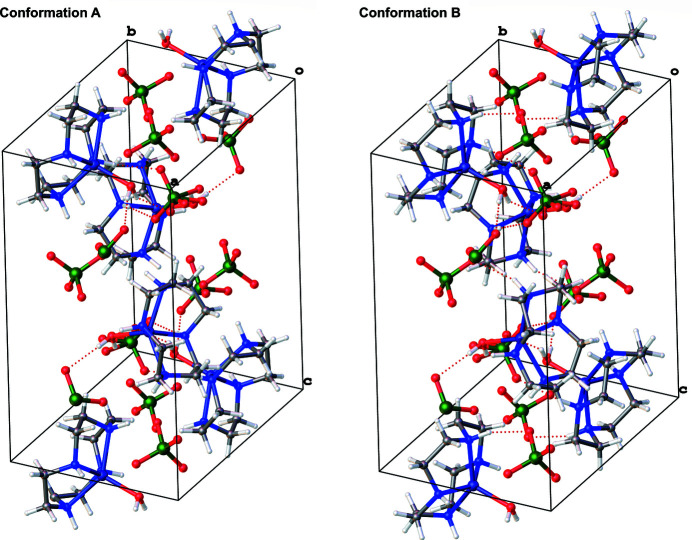
A view of the crystal packing of the title complex. Dashed lines denote the hydrogen bonds.

The two perchlorate ions are involved in inter­molecular hydrogen bonds with the cationic Zn^II^ complex (Table 1 ▸). In the crystal, inter­molecular hydrogen-bonding inter­actions connect neighboring mol­ecules, forming a three-dimensional network (Fig. 2 ▸). As far as we know, an aqua­(cyclen)copper(II) complex has already been reported (Pérez-Toro *et al.*, 2015 ▸), but the aqua­(cyclen)zinc(II) complex has not. The title aqua­(cyclen)zinc(II) complex has been well studied as Zn^II^-containing enzyme models, such as alkaline phosphatase, β-lactamase, and carbonic anhydrase, to elucidate the essential roles of Zn^II^ (Kimura *et al.*, 1995 ▸; Kitajima *et al.*, 1993 ▸; Zhang *et al.*, 1993 ▸; Zhang & van Eldik, 1995 ▸). We succeeded in determining its crystal structure at this time.

## Synthesis and crystallization

The title complex was prepared as fine white solid according to a previously reported method (Koike *et al.*, 1994 ▸) and then crystallized from aqueous ethanol.

**Caution!** Perchlorate salts of metal complexes with organic ligands are potentially explosive. Only small amounts of material should be prepared, and these should be handled with care.

## Refinement

Crystal data, data collection and structure refinement details are summarized in Table 2 ▸. In the final cycles of refinement, 12 outliers were omitted.

## Supplementary Material

Crystal structure: contains datablock(s) I. DOI: 10.1107/S2414314621003977/vm4048sup1.cif

Structure factors: contains datablock(s) I. DOI: 10.1107/S2414314621003977/vm4048Isup2.hkl

CCDC reference: 2067247

Additional supporting information:  crystallographic information; 3D view; checkCIF report

## Figures and Tables

**Table 1:** Hydrogen-bond geometry (Å, °)

*D*—H⋯*A*	*D*—H	H⋯*A*	*D*⋯*A*	*D*—H⋯*A*
O1—H1*A*⋯O9*A*	0.86	2.48	3.12 (3)	132
O1—H1*A*⋯O9*B*	0.86	1.94	2.68 (4)	145
O1—H1*B*⋯O6	0.85	2.06	2.914 (9)	173
O1—H1*B*⋯O7	0.85	2.54	3.088 (7)	123
N2*A*—H2*A*⋯O7	0.98	2.37	3.144 (12)	135
N2*B*—H2*B*⋯O4^i^	0.98	2.49	3.086 (11)	119
N3*A*—H3*A*⋯O2^ii^	0.98	2.59	3.312 (10)	130
N3*B*—H3*B*⋯O2^ii^	0.98	2.47	3.170 (12)	128
N4*A*—H4*A*⋯O5^iii^	0.98	2.18	3.094 (9)	155
N4*A*—H4*A*⋯O8^iii^	0.98	2.49	3.103 (10)	120
N4*B*—H4*B*⋯O5^iii^	0.98	2.1	3.030 (11)	157
N1*A*—H1*AA*⋯O8	0.98	2.15	3.099 (10)	162
N1*B*—H1*BA*⋯O8	0.98	2.16	3.105 (13)	163

Symmetry codes: (i) 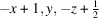 ; (ii) 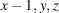 ; (iii) 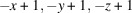 .

**Table 2:** Experimental details

Crystal data	
Chemical formula	[Zn(C_8_H_20_N_4_)(H_2_O)](ClO_4_)_2_
*M*_r_	454.56
Crystal system, space group	Monoclinic, *P*2/*c*
Temperature (K)	93
*a*, *b*, *c* (Å)	12.3428 (6), 8.4603 (4), 16.0543 (6)
β (°)	92.881 (4)
*V* (Å^3^)	1674.33 (13)
*Z*	4
Radiation type	Cu *K*α
μ (mm^−1^)	5.48
Crystal size (mm)	0.29 × 0.16 × 0.04

Data collection	
Diffractometer	Rigaku Synergy-i
Absorption correction	Gaussian (*CrysAlis PRO*; Rigaku OD, 2020 ▸)
*T*_min_, *T*_max_	0.535, 1.000
No. of measured, independent and observed [*I* > 2σ(*I*)] reflections	7740, 3025, 2670
*R*_int_	0.057
(sin θ/λ)_max_ (Å^−1^)	0.603

Refinement	
*R*[*F* ^2^ > 2σ(*F* ^2^)], *wR*(*F* ^2^), *S*	0.067, 0.186, 1.08
No. of reflections	3025
No. of parameters	301
H-atom treatment	H-atom parameters constrained
Δρ_max_, Δρ_min_ (e Å^−3^)	1.15, −0.84

Computer programs: *CrysAlis PRO* (Rigaku OD, 2020 ▸), *SHELXT* (Sheldrick, 2015*a* ▸), *SHELXL* (Sheldrick, 2015*b* ▸) and *OLEX2* (Dolomanov *et al.*, 2009 ▸).

## full crystallographic data

### Crystal data

**Table:**

[Zn(C_8_H_20_N_4_)(H_2_O)](ClO_4_)_2_	*F*(000) = 936
*M**_r_* = 454.56	*D*_x_ = 1.803 Mg m^−^^3^
Monoclinic, *P*2/*c*	Cu *K*α radiation, λ = 1.54184 Å
*a* = 12.3428 (6) Å	Cell parameters from 3951 reflections
*b* = 8.4603 (4) Å	θ = 5.5–68.1°
*c* = 16.0543 (6) Å	µ = 5.48 mm^−^^1^
β = 92.881 (4)°	*T* = 93 K
*V* = 1674.33 (13) Å^3^	Block, clear light colourless
*Z* = 4	0.29 × 0.16 × 0.04 mm

### Data collection

**Table:**

Rigaku_Synergy-i diffractometer	3025 independent reflections
Radiation source: micro-focus sealed X-ray tube, PhotonJet (Cu) X-ray Source	2670 reflections with *I* > 2σ(*I*)
Mirror monochromator	*R*_int_ = 0.057
Detector resolution: 10.0000 pixels mm^-1^	θ_max_ = 68.4°, θ_min_ = 3.6°
ω scans	*h* = −14→14
Absorption correction: gaussian (CrysAlisPro; Rigaku OD, 2020)	*k* = −10→9
*T*_min_ = 0.535, *T*_max_ = 1.000	*l* = −19→8
7740 measured reflections	

### Refinement

**Table:**

Refinement on *F*^2^	Primary atom site location: dual
Least-squares matrix: full	Hydrogen site location: mixed
*R*[*F*^2^ > 2σ(*F*^2^)] = 0.067	H-atom parameters constrained
*wR*(*F*^2^) = 0.186	*w* = 1/[σ^2^(*F*_o_^2^) + (0.0991*P*)^2^ + 7.9372*P*] where *P* = (*F*_o_^2^ + 2*F*_c_^2^)/3
*S* = 1.08	(Δ/σ)_max_ < 0.001
3025 reflections	Δρ_max_ = 1.15 e Å^−^^3^
301 parameters	Δρ_min_ = −0.84 e Å^−^^3^
0 restraints	

### Special details

**Table:**

Geometry. All esds (except the esd in the dihedral angle between two l.s. planes) are estimated using the full covariance matrix. The cell esds are taken into account individually in the estimation of esds in distances, angles and torsion angles; correlations between esds in cell parameters are only used when they are defined by crystal symmetry. An approximate (isotropic) treatment of cell esds is used for estimating esds involving l.s. planes.
Refinement. All hydrogen atoms were placed on calculated positions and refined in riding mode, with *U*_iso_(H) values assigned as 1.2*U*_eq_ of the parent atoms (1.5 times for water molecule O1).

### Fractional atomic coordinates and isotropic or equivalent isotropic displacement parameters (Å^2^)

**Table:**

	*x*	*y*	*z*	*U*_iso_*/*U*_eq_	Occ. (<1)
Zn1	0.26041 (6)	0.65100 (8)	0.40586 (4)	0.0237 (3)	
Cl1	0.77745 (10)	0.74888 (14)	0.44398 (7)	0.0253 (3)	
Cl3	0.500000	0.3592 (2)	0.250000	0.0379 (5)	
Cl2	0.000000	0.4021 (3)	0.250000	0.0427 (5)	
O2	0.8653 (4)	0.6503 (5)	0.4747 (3)	0.0376 (10)	
O1	0.2571 (4)	0.4307 (5)	0.3658 (3)	0.0380 (10)	
H1A	0.288342	0.423897	0.319539	0.057*	
H1B	0.191532	0.403407	0.353540	0.057*	
O5	0.6950 (4)	0.6530 (4)	0.4022 (2)	0.0354 (10)	
O4	0.8158 (4)	0.8651 (5)	0.3871 (3)	0.0386 (10)	
O3	0.7314 (4)	0.8284 (5)	0.5133 (3)	0.0450 (11)	
O8	0.5265 (5)	0.4556 (6)	0.3200 (3)	0.0565 (14)	
O7	0.0864 (5)	0.4984 (7)	0.2230 (3)	0.0672 (17)	
C6	0.1725 (5)	0.7292 (7)	0.5681 (3)	0.0337 (13)	
H6AA	0.166719	0.628890	0.596708	0.040*	0.5
H6AB	0.130034	0.806911	0.596576	0.040*	0.5
H6BC	0.133141	0.634016	0.581254	0.040*	0.5
H6BD	0.182279	0.791458	0.618632	0.040*	0.5
O9A	0.430 (3)	0.243 (3)	0.2710 (8)	0.066 (6)	0.62 (7)
C8	0.4612 (5)	0.7428 (8)	0.5014 (4)	0.0414 (15)	
H8AA	0.487825	0.803166	0.549486	0.050*	0.5
H8AB	0.512480	0.658178	0.492711	0.050*	0.5
H8BC	0.524175	0.810202	0.510715	0.050*	0.5
H8BD	0.479795	0.636990	0.520513	0.050*	0.5
C4	0.0710 (5)	0.8437 (8)	0.3503 (4)	0.0392 (14)	
H4AA	0.018566	0.760976	0.337036	0.047*	0.5
H4AB	0.043655	0.941480	0.325667	0.047*	0.5
H4BC	0.031273	0.783663	0.307339	0.047*	0.5
H4BD	0.023943	0.927003	0.368971	0.047*	0.5
C2	0.3599 (6)	0.8620 (7)	0.2838 (4)	0.0377 (14)	
H2AA	0.351470	0.797633	0.233856	0.045*	0.5
H2AB	0.412558	0.944234	0.274066	0.045*	0.5
H2BC	0.399202	0.791877	0.248175	0.045*	0.5
H2BD	0.352000	0.963274	0.255853	0.045*	0.5
N4A	0.3533 (8)	0.6720 (10)	0.5194 (5)	0.0214 (17)	0.5
H4A	0.362285	0.569147	0.546981	0.026*	0.5
N2A	0.1762 (9)	0.8030 (13)	0.3149 (5)	0.0256 (18)	0.5
H2A	0.163507	0.746012	0.262159	0.031*	0.5
N1A	0.3988 (8)	0.7609 (10)	0.3570 (6)	0.0238 (18)	0.5
H1AA	0.446853	0.678514	0.336943	0.029*	0.5
N3A	0.1304 (7)	0.7131 (10)	0.4792 (7)	0.0243 (18)	0.5
H3A	0.074948	0.630179	0.475250	0.029*	0.5
C3A	0.2519 (10)	0.9354 (13)	0.3034 (6)	0.028 (2)	0.5
H3AA	0.259465	0.998537	0.353821	0.034*	0.5
H3AB	0.225447	1.002771	0.257917	0.034*	0.5
C5A	0.0848 (9)	0.8626 (12)	0.4440 (8)	0.025 (2)	0.5
H5AA	0.133508	0.949844	0.457747	0.030*	0.5
H5AB	0.015347	0.884647	0.467187	0.030*	0.5
C7A	0.2892 (10)	0.7804 (15)	0.5691 (7)	0.027 (2)	0.5
H7AA	0.293554	0.886458	0.546567	0.033*	0.5
H7AB	0.319046	0.782356	0.626111	0.033*	0.5
C1A	0.4564 (10)	0.8461 (13)	0.4278 (7)	0.029 (2)	0.5
H1AB	0.529179	0.873893	0.413020	0.034*	0.5
H1AC	0.417900	0.942758	0.439999	0.034*	0.5
N1B	0.4265 (8)	0.7403 (14)	0.4110 (8)	0.034 (2)	0.5
H1BA	0.471976	0.663912	0.382960	0.041*	0.5
C1B	0.4205 (10)	0.8823 (15)	0.3617 (8)	0.037 (3)	0.5
H1BB	0.386928	0.964996	0.393325	0.045*	0.5
H1BC	0.493463	0.916393	0.350715	0.045*	0.5
N4B	0.2823 (11)	0.6851 (12)	0.5371 (6)	0.033 (2)	0.5
H4B	0.308024	0.586973	0.563809	0.040*	0.5
C7B	0.3640 (11)	0.8079 (15)	0.5489 (7)	0.037 (3)	0.5
H7BA	0.383889	0.822821	0.607646	0.044*	0.5
H7BB	0.338491	0.907531	0.525404	0.044*	0.5
N3B	0.1035 (9)	0.7343 (14)	0.4242 (7)	0.039 (3)	0.5
H3B	0.052515	0.645734	0.426759	0.047*	0.5
C5B	0.1084 (10)	0.8221 (16)	0.5035 (8)	0.037 (3)	0.5
H5BA	0.035586	0.839754	0.521684	0.044*	0.5
H5BB	0.142278	0.924175	0.495850	0.044*	0.5
N2B	0.2492 (10)	0.7937 (12)	0.2972 (6)	0.033 (2)	0.5
H2B	0.224070	0.730603	0.248759	0.039*	0.5
C3B	0.1686 (14)	0.914 (2)	0.3155 (7)	0.044 (3)	0.5
H3BA	0.147684	0.971106	0.264677	0.053*	0.5
H3BB	0.200630	0.989686	0.355062	0.053*	0.5
O9B	0.384 (3)	0.296 (5)	0.255 (3)	0.066 (12)	0.38 (7)
O6	0.0412 (6)	0.3127 (13)	0.3175 (6)	0.138 (5)	

### Atomic displacement parameters (Å^2^)

**Table:**

	*U*^11^	*U*^22^	*U*^33^	*U*^12^	*U*^13^	*U*^23^
Zn1	0.0349 (4)	0.0181 (4)	0.0184 (4)	−0.0015 (3)	0.0045 (3)	−0.0025 (2)
Cl1	0.0352 (7)	0.0198 (6)	0.0207 (6)	−0.0012 (5)	0.0004 (5)	−0.0014 (4)
Cl3	0.0561 (13)	0.0208 (9)	0.0381 (11)	0.000	0.0146 (9)	0.000
Cl2	0.0591 (13)	0.0369 (11)	0.0334 (10)	0.000	0.0138 (9)	0.000
O2	0.041 (2)	0.033 (2)	0.039 (2)	0.0041 (18)	0.0003 (18)	0.0095 (17)
O1	0.052 (3)	0.024 (2)	0.040 (2)	−0.0067 (19)	0.0189 (19)	−0.0098 (17)
O5	0.049 (3)	0.023 (2)	0.032 (2)	−0.0112 (17)	−0.0131 (18)	0.0006 (15)
O4	0.058 (3)	0.029 (2)	0.029 (2)	−0.0082 (19)	0.0013 (18)	0.0091 (16)
O3	0.056 (3)	0.041 (2)	0.039 (2)	−0.003 (2)	0.018 (2)	−0.0173 (19)
O8	0.071 (3)	0.031 (2)	0.064 (3)	0.009 (2)	−0.020 (3)	−0.017 (2)
O7	0.089 (4)	0.059 (3)	0.057 (3)	−0.030 (3)	0.036 (3)	−0.019 (3)
C6	0.054 (4)	0.026 (3)	0.023 (3)	0.003 (3)	0.016 (2)	−0.003 (2)
O9A	0.089 (13)	0.052 (8)	0.053 (6)	−0.048 (8)	−0.021 (6)	0.024 (6)
C8	0.042 (3)	0.050 (4)	0.032 (3)	0.004 (3)	−0.003 (3)	−0.013 (3)
C4	0.042 (3)	0.038 (3)	0.036 (3)	0.008 (3)	−0.008 (3)	−0.010 (3)
C2	0.056 (4)	0.032 (3)	0.027 (3)	−0.004 (3)	0.014 (3)	0.007 (2)
N4A	0.026 (5)	0.015 (4)	0.024 (4)	0.008 (4)	0.003 (4)	0.000 (3)
N2A	0.039 (6)	0.021 (6)	0.017 (4)	−0.007 (5)	0.003 (4)	−0.004 (4)
N1A	0.030 (5)	0.016 (5)	0.026 (5)	0.002 (4)	0.003 (4)	0.001 (4)
N3A	0.030 (5)	0.015 (5)	0.028 (5)	0.004 (4)	0.000 (4)	−0.006 (4)
C3A	0.042 (7)	0.027 (6)	0.016 (5)	−0.001 (5)	−0.003 (4)	−0.002 (4)
C5A	0.023 (5)	0.016 (5)	0.036 (7)	0.003 (4)	0.000 (4)	−0.006 (4)
C7A	0.036 (7)	0.027 (6)	0.019 (5)	0.004 (5)	0.000 (5)	−0.001 (5)
C1A	0.039 (6)	0.013 (5)	0.035 (7)	−0.012 (5)	0.009 (5)	−0.008 (4)
N1B	0.022 (5)	0.044 (8)	0.037 (6)	0.005 (5)	0.002 (4)	−0.001 (5)
C1B	0.040 (7)	0.035 (7)	0.038 (7)	−0.014 (5)	0.018 (5)	−0.007 (5)
N4B	0.068 (9)	0.013 (5)	0.019 (5)	0.011 (5)	0.000 (5)	0.002 (4)
C7B	0.046 (8)	0.035 (7)	0.028 (6)	0.007 (6)	−0.009 (5)	−0.008 (5)
N3B	0.035 (6)	0.046 (7)	0.037 (7)	−0.010 (5)	0.010 (5)	−0.011 (5)
C5B	0.037 (6)	0.036 (7)	0.038 (7)	−0.005 (5)	0.013 (5)	−0.005 (6)
N2B	0.052 (7)	0.028 (5)	0.018 (4)	0.006 (5)	−0.002 (4)	−0.002 (4)
C3B	0.072 (12)	0.035 (9)	0.024 (6)	0.008 (7)	−0.014 (6)	−0.002 (5)
O9B	0.071 (17)	0.073 (17)	0.056 (18)	−0.027 (15)	0.028 (13)	−0.040 (13)
O6	0.074 (5)	0.188 (10)	0.156 (8)	0.037 (6)	0.033 (5)	0.136 (8)

### Geometric parameters (Å, º)

**Table:**

Zn1—O1	1.971 (4)	C6—C7A	1.503 (14)
Zn1—N4A	2.111 (9)	C6—N4B	1.513 (14)
Zn1—N2A	2.171 (10)	C6—C5B	1.495 (15)
Zn1—N1A	2.129 (9)	C8—N4A	1.501 (12)
Zn1—N3A	2.104 (9)	C8—C1A	1.468 (13)
Zn1—N1B	2.183 (10)	C8—N1B	1.492 (13)
Zn1—N4B	2.130 (10)	C8—C7B	1.555 (15)
Zn1—N3B	2.096 (11)	C4—N2A	1.484 (13)
Zn1—N2B	2.121 (9)	C4—C5A	1.514 (13)
Cl1—O2	1.436 (4)	C4—N3B	1.542 (14)
Cl1—O5	1.440 (4)	C4—C3B	1.479 (19)
Cl1—O4	1.438 (4)	C2—N1A	1.512 (12)
Cl1—O3	1.442 (4)	C2—C3A	1.519 (14)
Cl3—O8^i^	1.413 (5)	C2—C1B	1.435 (15)
Cl3—O8	1.413 (5)	C2—N2B	1.510 (14)
Cl3—O9A	1.364 (14)	N4A—C7A	1.472 (14)
Cl3—O9A^i^	1.364 (14)	N2A—C3A	1.476 (15)
Cl3—O9B	1.53 (3)	N1A—C1A	1.496 (15)
Cl3—O9B^i^	1.53 (3)	N3A—C5A	1.485 (14)
Cl2—O7^ii^	1.427 (5)	N1B—C1B	1.439 (17)
Cl2—O7	1.427 (5)	N4B—C7B	1.454 (18)
Cl2—O6^ii^	1.397 (7)	N3B—C5B	1.473 (16)
Cl2—O6	1.397 (7)	N2B—C3B	1.466 (18)
C6—N3A	1.500 (12)		
			
O1—Zn1—N4A	111.3 (3)	O6—Cl2—O7	107.3 (5)
O1—Zn1—N2A	109.8 (3)	O6—Cl2—O6^ii^	114.4 (11)
O1—Zn1—N1A	107.2 (3)	N3A—C6—C7A	108.8 (6)
O1—Zn1—N3A	114.5 (3)	C5B—C6—N4B	110.7 (7)
O1—Zn1—N1B	110.1 (3)	C1A—C8—N4A	113.1 (7)
O1—Zn1—N4B	116.8 (3)	N1B—C8—C7B	107.0 (7)
O1—Zn1—N3B	111.1 (3)	N2A—C4—C5A	110.4 (7)
O1—Zn1—N2B	105.7 (3)	C3B—C4—N3B	110.4 (7)
N4A—Zn1—N2A	138.7 (3)	N1A—C2—C3A	108.5 (6)
N4A—Zn1—N1A	82.6 (4)	C1B—C2—N2B	111.0 (7)
N1A—Zn1—N2A	81.9 (4)	C8—N4A—Zn1	108.4 (5)
N3A—Zn1—N4A	83.8 (4)	C7A—N4A—Zn1	103.7 (7)
N3A—Zn1—N2A	82.9 (4)	C7A—N4A—C8	111.2 (8)
N3A—Zn1—N1A	138.2 (3)	C4—N2A—Zn1	106.2 (5)
N4B—Zn1—N1B	81.0 (5)	C3A—N2A—Zn1	104.4 (7)
N3B—Zn1—N1B	138.7 (4)	C3A—N2A—C4	116.3 (10)
N3B—Zn1—N4B	83.6 (5)	C2—N1A—Zn1	107.7 (6)
N3B—Zn1—N2B	84.4 (5)	C1A—N1A—Zn1	106.8 (7)
N2B—Zn1—N1B	81.8 (5)	C1A—N1A—C2	116.0 (8)
N2B—Zn1—N4B	137.4 (4)	C6—N3A—Zn1	108.5 (5)
O2—Cl1—O5	109.7 (3)	C5A—N3A—Zn1	106.5 (7)
O2—Cl1—O4	110.4 (3)	C5A—N3A—C6	113.0 (8)
O2—Cl1—O3	109.0 (3)	N2A—C3A—C2	106.4 (9)
O5—Cl1—O3	109.0 (3)	N3A—C5A—C4	108.1 (8)
O4—Cl1—O5	109.7 (2)	N4A—C7A—C6	110.8 (10)
O4—Cl1—O3	109.1 (3)	C8—C1A—N1A	108.8 (9)
O8—Cl3—O8^i^	109.5 (4)	C8—N1B—Zn1	105.3 (6)
O8^i^—Cl3—O9B	94 (3)	C1B—N1B—Zn1	104.2 (8)
O8—Cl3—O9B	109.6 (8)	C1B—N1B—C8	121.9 (10)
O8—Cl3—O9B^i^	94 (3)	C2—C1B—N1B	112.9 (10)
O8^i^—Cl3—O9B^i^	109.6 (8)	C6—N4B—Zn1	106.7 (6)
O9A—Cl3—O8	110.2 (8)	C7B—N4B—Zn1	106.2 (8)
O9A^i^—Cl3—O8	119.3 (9)	C7B—N4B—C6	114.0 (10)
O9A^i^—Cl3—O8^i^	110.2 (8)	N4B—C7B—C8	103.3 (9)
O9A—Cl3—O8^i^	119.3 (9)	C4—N3B—Zn1	107.4 (6)
O9A^i^—Cl3—O9A	88 (3)	C5B—N3B—Zn1	107.0 (8)
O9A^i^—Cl3—O9B^i^	29.6 (13)	C5B—N3B—C4	111.1 (10)
O9A—Cl3—O9B^i^	112 (3)	N3B—C5B—C6	109.3 (11)
O7—Cl2—O7^ii^	110.4 (5)	C2—N2B—Zn1	108.2 (6)
O6^ii^—Cl2—O7^ii^	107.3 (5)	C3B—N2B—Zn1	104.4 (8)
O6^ii^—Cl2—O7	108.8 (5)	C3B—N2B—C2	113.0 (10)
O6—Cl2—O7^ii^	108.8 (5)	N2B—C3B—C4	111.6 (13)

Symmetry codes: (i) −*x*+1, *y*, −*z*+1/2; (ii) −*x*, *y*, −*z*+1/2.

### Hydrogen-bond geometry (Å, º)

**Table:**

*D*—H···*A*	*D*—H	H···*A*	*D*···*A*	*D*—H···*A*
O1—H1*A*···O9*A*	0.86	2.48	3.12 (3)	132
O1—H1*A*···O9*B*	0.86	1.94	2.68 (4)	145
O1—H1*B*···O6	0.85	2.06	2.914 (9)	173
O1—H1*B*···O7	0.85	2.54	3.088 (7)	123
N2*A*—H2*A*···O7	0.98	2.37	3.144 (12)	135
N2*B*—H2*B*···O4^i^	0.98	2.49	3.086 (11)	119
N3*A*—H3*A*···O2^iii^	0.98	2.59	3.312 (10)	130
N3*B*—H3*B*···O2^iii^	0.98	2.47	3.170 (12)	128
N4*A*—H4*A*···O5^iv^	0.98	2.18	3.094 (9)	155
N4*A*—H4*A*···O8^iv^	0.98	2.49	3.103 (10)	120
N4*B*—H4*B*···O5^iv^	0.98	2.1	3.030 (11)	157
N1*A*—H1*AA*···O8	0.98	2.15	3.099 (10)	162
N1*B*—H1*BA*···O8	0.98	2.16	3.105 (13)	163

Symmetry codes: (i) −*x*+1, *y*, −*z*+1/2; (iii) *x*−1, *y*, *z*; (iv) −*x*+1, −*y*+1, −*z*+1.
